# A *Pig-a* conditional knock-out mice model mediated by Vav-iCre: stable GPI-deficient and mild hemolysis

**DOI:** 10.1186/s40164-022-00254-5

**Published:** 2022-01-15

**Authors:** Yingying Chen, Hui Liu, Lijie Zeng, Liyan Li, Dan Lu, Zhaoyun Liu, Rong Fu

**Affiliations:** Department of Hematology, Tianjin Medical University General Hospital, 154 Anshan Street, Heping District, Tianjin, 300052 People’s Republic of China

**Keywords:** Paroxysmal nocturnal hemoglobinuria, *Pig-a* gene, Mouse model, Conditional knock-out

## Abstract

Paroxysmal nocturnal hemoglobinuria is a clonal disease caused by *PIG-A* mutation of hematopoietic stem cells. At present, there is no suitable PNH animal model for basic research, therefore, it is urgent to establish a stable animal model. We constructed a *Pig-a* conditional knock-out mice model by ES targeting technique and Vav-iCre. The expressions of GPI and GPI-AP were almost completely absent in CKO homozygote mice, and the proportion of the deficiency remained stable from birth. In CKO heterozygote mice, the proportion of the deficiency of GPI and GPI-AP was partially absent and decreased gradually from birth until it reached a stable level at 3 months after birth and remained there for life. Compared with normal C57BL/6N mice and Flox mice, pancytopenia was found in CKO homozygous mice, and leukopenia and anemia were found in CKO heterozygotes mice. Meanwhile, in CKO mice, the serum LDH, TBIL, IBIL, complement C5b-9 levels were increased, and the concentration of plasma FHb was increased. Hemosiderin granulosa cells can be seen more easily in the spleens of CKO mice. What’s more, CKO mice had stable transcription characteristics. In conclusion, our mouse model has stable GPI-deficient and mild hemolysis, which may be an ideal in vivo experimental model for PNH.

**Supplementary Information:**

The online version contains supplementary material available at 10.1186/s40164-022-00254-5.

## Key points

1. Constructed a mouse model of *Pig-a* gene knockout in hematopoietic system mediated by Vav-iCre.
2. The mouse model was verified to have stable GPI-deficient and mild hemolysis.
3. The mouse model have stable transcription characteristics.

## Introduction

Paroxysmal nocturnal hemoglobinuria (PNH) is a disease caused by defect of hematopoietic stem cell membrane caused by acquired somatic mutation [1]. The molecular pathogenesis of PNH is the mutation of phosphatidylinositol glycan-class A (*PIG-A*) gene on the X chromosome of hematopoietic stem cells, which leads to the disorder of glycophosphatidylinositol (GPI) synthesis [2], resulting in the deficiency of glycosylphosphatidylinositol anchor protein (GPI-AP) on the cell membrane surface [3–7]. The main clinical manifestations of PNH are hemolytic anemia, thrombophilia and bone marrow failure.

At present, there is no suitable PNH animal model for basic research. It has been reported that most of the genome-wide knockout (KO) mice die in utero, and the proportion of GPI-AP deficient blood cells in the surviving mice is very low (about 10–30%) [8], and further decreases with the prolongation of postnatal time [9]. After that, *Pig-a* gene conditional Knockout (CKO) mice were constructed using Cre/loxP technology to obtain mice with higher chimerism rate and higher proportion of GPI-AP deficient (about 40–90%), and the proportion of GPI-AP deficient in CKO mice remained basically stable or slightly decreased after birth [10–13]. Tae-Hoon Shin et al. [14] used CRISPR/Cas9 technology to knockout the *PIG-A* gene of macaque hematopoietic stem cells, and then transplanted the edited hematopoietic stem cells back into macaque monkeys to obtain the PNH macaque model. The proportion of abnormal PNH clones in blood cells of the macaque model gradually decreased after birth and maintained at a certain level after birth. However, no increase in the proportion of PNH and no typical clinical manifestations such as anemia, hemoglobinuria and thrombosis were observed in either the mice models or the macaque models. As a result, the existing animal model of PNH cannot be really used in PNH related studies.

Vav-iCre mice express iCre in hematopoietic cells, endothelial cells, and testes [15]. When Vav-iCre is hybridized with mice containing the LoxP sequence, Cre mediated recombination results in the deletion of Flox sequence in the progeny hematopoietic cells, resulting in functional knockout of the target gene in the hematopoietic cells (and their progenitor cells). We construct a *Pig-a* CKO mice model mediated by Vav-iCre Through the detection of various indicators in mice, it was proved that this model not only had stable GPI deficient but also had the disease phenotype of mild intravascular hemolysis, which may be an ideal animal model for PNH and can be used in the related studies on the pathogenesis and treatment of PNH.

## Materials and methods

### Construction of Pig-a CKO mice

#### Construction of Flox mice

**Fig. 1:**
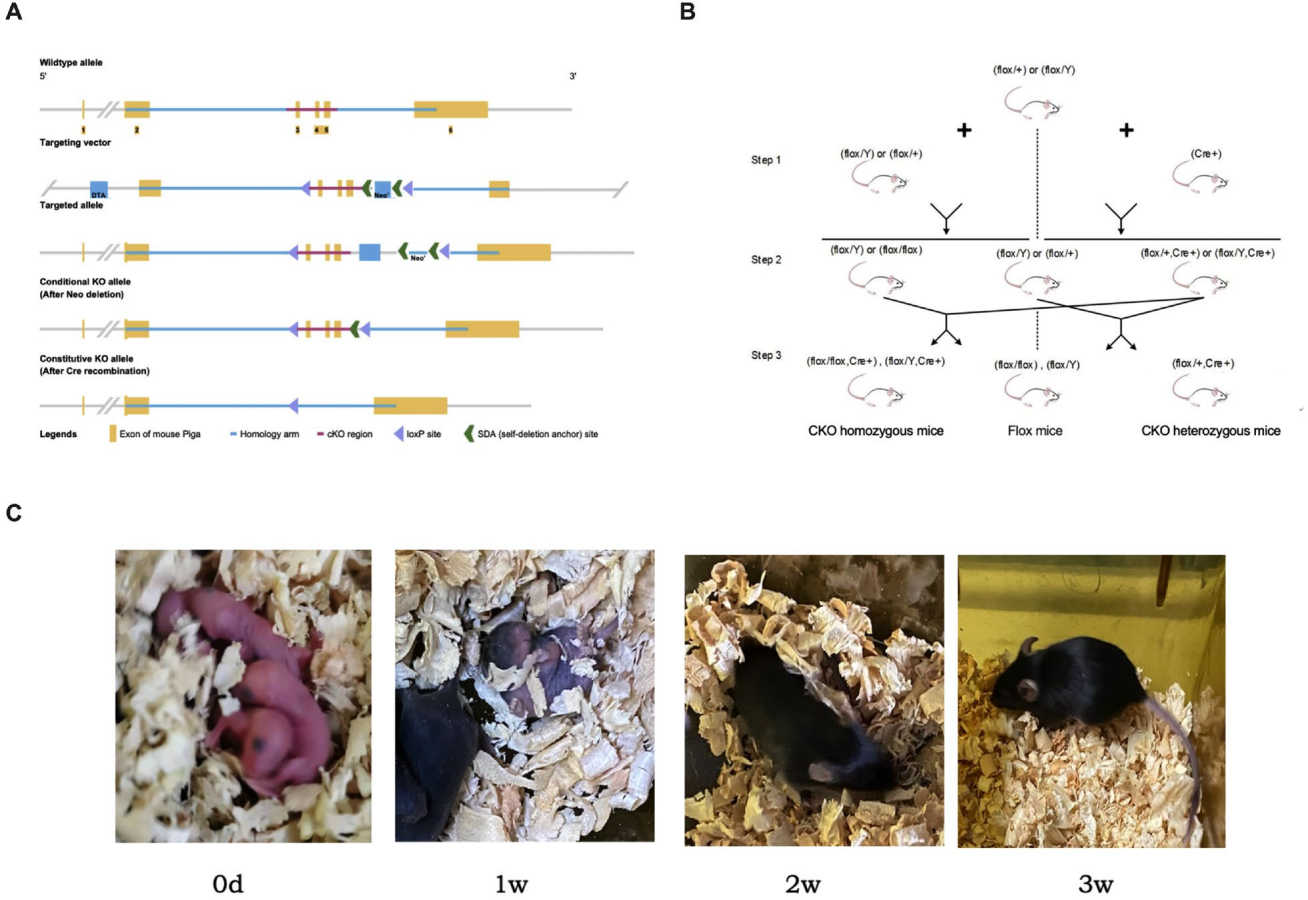
A schematic diagram of established a Pig-a CKO mice model mediated by Vav-iCre. **A** ES target strategy. **B** Propagation strategies. **C** Photos of offspring mice

LoxP was inserted on both sides of exon 3 and exon 5 of *Pig-a* gene to construct the target vector (Fig. 1A), and after verification, plasmid extraction and linearization were performed. The target vector was electrically transferred to embryonic stem cell (ESC) of C57BL/6N mice, and neomycin was added to select drug-resistant clones. The ESCs were verified by PCR, karyotype analysis and Southern Blot. The obtained positive ESCs were microinjected, and the injected blastocysts were transplanted into the pseudo-pregnant female mice. The offspring mice were numbered 1 week after birth, and the 1 cm tails of the mice to be tested were cut off 3 weeks after birth and genomic DNA was extracted with QIAamp DNA kits (Qiagen, 51304). *Pig-a* genotype was identified by PCR (primer sequences are shown in Table 1). The genotype of Flox homozygous female mice was Pig-a [Flox/Flox], and the genotype of Flox homozygous male mice was Pig-a [Flox/Y].

**Table 1:** Specific PCR primers of Pig-a, Vav-iCre

Name	Sequence (5′ to 3′)
Pia-a	Forward, GACTTCTGAACAAAATGAAGGCAGT
	Reverse, GTGCACAGCTGATTAGAAATCTAGG
Vav-iCre	Forward, GGTGTTGTAGTTGTCCCCACT
	Reverse, CAGGTTTTGGTGCACAGTCA

#### Propagation and genotype identification

Progeny mice were obtained by mating Flox mice with Vav-iCre mice (Fig. 1B). The offspring mice were numbered 1 week after birth, and the 1 cm tails of the mice to be tested were cut off 3 weeks after birth (Fig. 1C). Genomic DNA was extracted with QIAamp DNA kits (Qiagen, 51304). *Pig-a* and Vav-iCre genotype was identified by PCR (primer sequences are shown in Table 1). Female CKO homozygous genotype is *Pig-a* [Flox/Flox, Vav-iCre], male CKO homozygous genotype is *Pig-a* [Flox/Y, Vav-iCre], and female CKO heterozygous genotype is *Pig-a* [Flox/+, Vav-iCre]. All mice were fed in an individual ventilated cage (IVC) system (SPF grade). Follow-up experiments were divided into four groups: CKO homozygous mice, CKO heterozygous mice, Flox mice and normal C57BL/6N mice. The detection of disease-related indicators was divided into four groups with five mice in each group and the age difference was no more than 1 week.

### Validation of Pig-a CKO mice

#### Reverse transcription‑quantitative polymerase chain reaction (RT‑qPCR)

The mice were sacrificed, and the bone marrow cavities of both sides of femur were rinsed with PBS to obtain bone marrow cell suspension. The red blood cells were dissolved and centrifuged after PBS cleaning to obtain mouse bone marrow cells. RNeasy kit (Takara Bio, Inc.) was used to extracted total RNA from mice bone marrow cells. FastKing RT Kit (S8025, Tiangen) was used to synthesized cDNA from 1.5 μg total RNA. SuperReal PreMix Plus (SYBR Green) (S7717, Tiangen) and the Light Cycler 1.5 Real-Time PCR system (Roche Diagnostics, Indianapolis, USA) are used for RT-qPCR. Specific primers for amplification of Pig-a and Gapdh genes are detailed in Table 2. We used Bio-Rad large management software CFX 3.1 (Bio-Rad Labs, Inc.) to analyze melting and amplification curves and cycle threshold (Ct) values determined for every sample. The relative quantitative multiplier was expressed as 2^−ΔΔCt^ value, which was used for statistical analysis.

**Table 2:** Specific qRT-PCR primers of Pig-a, Gapdh

Name	Sequence (5′ to 3′)
Pig-a	Forward, TGTCCGTCATTCCTAACGCT
	Reverse, TCTTTGGTCCCTCTCCTCCAA
Gapdh	Forward, TGACCTCAACTACATGGTCTACA
	Reverse, CTTCCCATTCTCGGCCTTG

#### Western blot

After cell counting, 1 × 10^7^ mouse bone marrow cells were taken, and total protein was extracted by RIPA lysis method. Protein was separated by Tris-MOPS-SDS Running Buffer, 4–20% SurePAGE, electrotransferred to PVDF membranes by eBlot L1 Quick wet converter (L00686C, GenScript). PVDF membrane was placed in 5% occulting solution and closed in a shaker at room temperature for 1 h. After that, PVDF membrane was cut according to the Marker location and molecular weight of target protein, and the bands of PIG-A and GAPDH were immunoblotted with primary antibody against PIG-A (bs-9524R, Bioss) and GAPDH (D16H11, CST), followed by HRP-linked antibody (7074, CST). The image is exposed and stored using ECL developer and exposure instrument (GelView 6000Plus, BLT photonics technology).

### Examination of disease-related indicators

#### Flow cytometry

Flow cytometry was used to detect the expression of GPI and GPI-AP in peripheral blood cells at the age of 5w, 8w, 3m, 4m, 6m, 8m, 10m and 12m, respectively. RBC, B lymphocytes, T lymphocytes and granulocytes were labeled with BV421 Rat Anti-Mouse TER-119 (563998, BD), PE Rat Anti-Mouse CD45R/B220 (553090, BD), APC Hamster Anti-Mouse CD3e (553066, BD) and PerCP-Cy™5.5 Rat Anti-Mouse Gr-1 (552093, BD), respectively. We measured the expression of FLAER and APC Rat Anti-Mouse CD24 (562349, BD)/PE Hamster Anti-Mouse CD48 (557485, BD) on the surface of different blood cells. The samples were tested by BECKMAN COULTER. Twenty thousand cells were collected from each tube and the results were analyzed using CytExpert software.

#### Blood routine and blood biochemical tests of mice

Peripheral blood (about 50 μL) was collected into the EDTA anticoagulant EP tube for routine blood analysis with automatic routine blood analyzer (BC-2800Vet, mindray). 100 μL serum was taken for LDH, TBIL and IBIL analysis using an automatic blood biochemical analyzer (BS-240VET, mindray). Plasma free hemoglobin assay kit (Beijing Ruerda Biological Technology Co., Ltd) was used to detect the concentration of free hemoglobin (FHb) in mice plasma.

#### ELISA

Mice TCC C5b-9, C3a, C5a ELISA kit (SBJ-M0335, SBJ-H1735, SBJ-H1734, SenBeiJia Biological Technology Co., Ltd.) were used to detect the levels of serum complement C5b-9, C3a and C5a according to the protocol. Draw the standard curve according to the standard product concentration and calculate the sample pore concentration according to the standard curve.

#### Pathology

After the mice were sacrificed, one femur and spleen were removed and placed in 1.5 mL EP tubes preloaded with 4% paraformaldehyde, respectively. After paraffin embedding, slices were sliced (3–5 μm in thickness), the wax slices were attached to the slides, and baked in an oven (80 °C, 30–60 min). After HE staining, the tissue structure was observed under a microscope.

### Whole blood transcriptome sequencing analysis

Based on Illumina sequencing platform, we studied the differences of peripheral blood transcriptome levels in four groups of mice, and three 6-month-old mice were detected in each group. RNA-seq can be divided into two parts: building library sequencing and bioinformatics analysis.

#### Building library sequencing

Total amounts and integrity of RNA were assessed using the RNA Nano 6000 Assay Kit of the Bioanalyzer 2100 system (Agilent Technologies, CA, USA). mRNA was purified from total RNA by using poly-T oligo-attached magnetic beads. The mRNA was then randomly interrupted by divalent cations in the NEB Fragmentation Buffer, and the NEBNext^®^ Ultra™ RNA Library Prep Kit for Illumina^®^ Kit was used to build the Library. After the construction of the library, the library was initially quantified by Qubit2.0 Fluorometer, and qRT-PCR was used to accurately quantify the effective concentration of the library (the effective concentration of the library is higher than that of 2 nM) to ensure the quality of the library. After the library is qualified, the different libraries are pooling according to the effective concentration and the target amount of data off the machine, then being sequenced by the Illumina NovaSeq 6000. The end reading of 150 bp pairing is generated.

#### Bioinformatics analysis

The image data measured by the high-throughput sequencer are converted into sequence data (reads) by CASAVA base recognition. Clean data (clean reads) were obtained by removing reads containing adapter, reads containing N base and low-quality reads from raw data. All the downstream analyses were based on the clean data with high quality.

Clean reads after quality control were compared to the reference genome. Hisat2 (v2.0.5) software was used to quickly and accurately compare Clean Reads with the reference genome to obtain the location information of Reads on the reference genome. We used StringTie software for new transcript assembly. After the new transcript was assembled, database annotations such as Pfam, SUPERFAMILY, GO and KEGG were performed on the new transcript. Based on the location information of gene alignment on the reference genome, the number of reads covered by each gene (including the new prediction gene) from start to end was calculated using the Feature Recounts tool in Subread software. Reads with a comparison quality value of less than 10, unpaired reads, and reads to multiple regions of the genome were filtered out.

Differential expression analysis of two conditions/groups (two biological replicates per condition) was performed using the DESeq2 R package (1.20.0). The resulting P-values were adjusted using the Benjamini and Hochberg’s approach. Padj ≤ 0.05 and |log2 (foldchange)| ≥ 1 were set as the threshold for significantly differential expression. Gene Ontology (GO) enrichment analysis, KEGG were used for enrichment analysis of differentially expressed genes independently, and corrected P value less than 0.05 was considered to be significantly enriched.

### Statistical analysis

GraphPad Prism 8.2.1 software was used for statistical analysis. Quantitative data satisfying normal distribution and homogeneity of variance were expressed as mean ± standard deviation. Shapiro–Wilk test was used to test the normality of multiple groups of data, and *p* > 0.05 was considered as conforming to normality. Chi-square test was used for comparison of multiple groups of data consistent with normal distribution and homogeneous variance, and Holm-Sidak's multiple t test was used for comparison between groups. Kruskal–Wallis test was used for the comparison of multiple groups of data with variances inconsistent or with normal distribution, and Dunn's multiple t-test was used for the comparison between groups. *p* < 0.05 was considered to indicate a statistically significant difference.

## Results

### *Pig-a* gene is knock-out in CKO mice

**Fig. 2:**
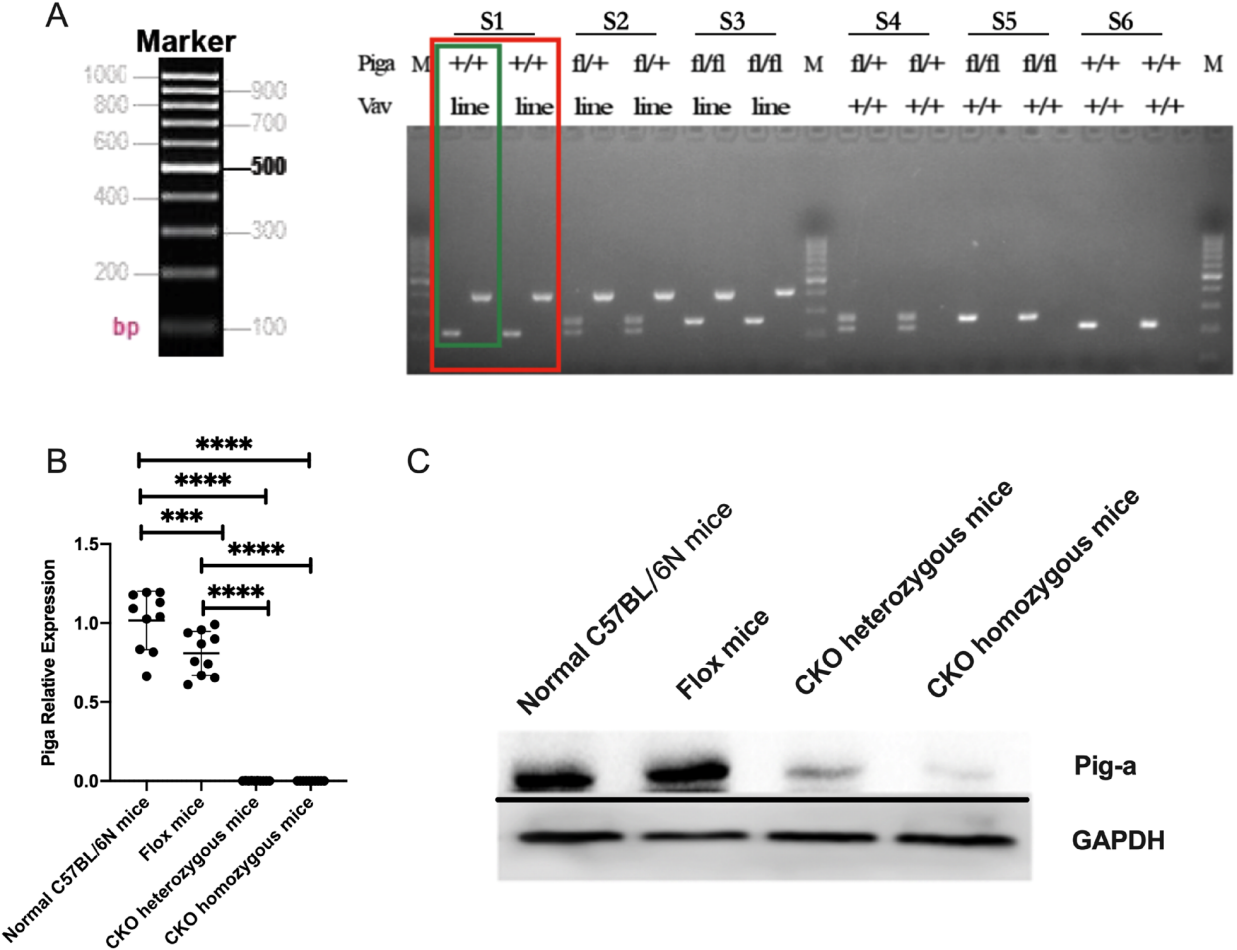
The expression level of *Pig-a.* **A** Identification of mice genotypes. One mice sample were repeated twice (red frame), and each sample (green frame) was used to identify LoxP in the left hole and Cre in the right hole. (S1: Vav-iCre mouse; S2: CKO heterozygote mouse; S3: CKO homozygote mouse; S4: Flox heterozygote mouse; S5: Flox homozygote mouse; S6: Normal C57BL/6N mouse); **B** mRNA expression level of *Pig-a* in mouse bone marrow cells (*p < 0.05, **p < 0.01, ***p < 0.001, ****p < 0.0001). C Protein expression level of Pig-a in mouse bone marrow cells

PCR results showed that normal C57BL/6N mice and Vav-iCre mice could amplify a 216 bp normal *Pig-a* fragment, while Flox homozygous and CKO homozygous mice could amplify a 256 bp mutant *Pig-a* fragment. A 216 pb normal fragment and a 256 bp mutant fragment were amplified from Flox heterozygotes and CKO heterozygotes. Normal C57BL/6N mice and Flox mice did not express Vav-iCre, and there was no band after amplification. Vav-iCre mice and CKO mice could amplify the target fragment with the size of 390 bp (Fig. 2A).

*Pig-a* mRNA relative expression level in bone marrow cells of normal C57BL/6N mice, Flox mice, CKO heterozygous mice and CKO homozygous mice were 1.017 ± 0.1850, 0.8082 ± 0.1392, 0.00098 ± 0.00038 and 0.000047 ± 0.0002654 (*p* < 0.0001), in which the level of *Pig-a* mRNA in CKO heterozygous mice and CKO homozygous mice were significantly lower than those of normal C57BL/6N mice and Flox mice (*p* < 0.0001). Compared with normal C57BL/6N mice, the mRNA relative expression level of *Pig-a* in Flox mice bone marrow cells were lower (*p* = 0.0005) (Fig. 2B). Meanwhile, compared with normal C57BL/6N mice, the protein expression level of Pig-a in CKO heterozygous mice was significantly reduced, and Pig-a protein expression in CKO homozygous mice was almost non-expression, but the protein expression level of Pig-a in Flox mice was unchanged (Fig. 2C).

After genotype identification, Flox mice, CKO heterozygous and CKO heterozygous were separated into cages. The body weight, survival and urine color of the mice were monitored regularly. There was no significant difference between four groups.

### The expressions of GPI and GPI-AP were almost completely absent in CKO homozygote mice

**Fig. 3:**
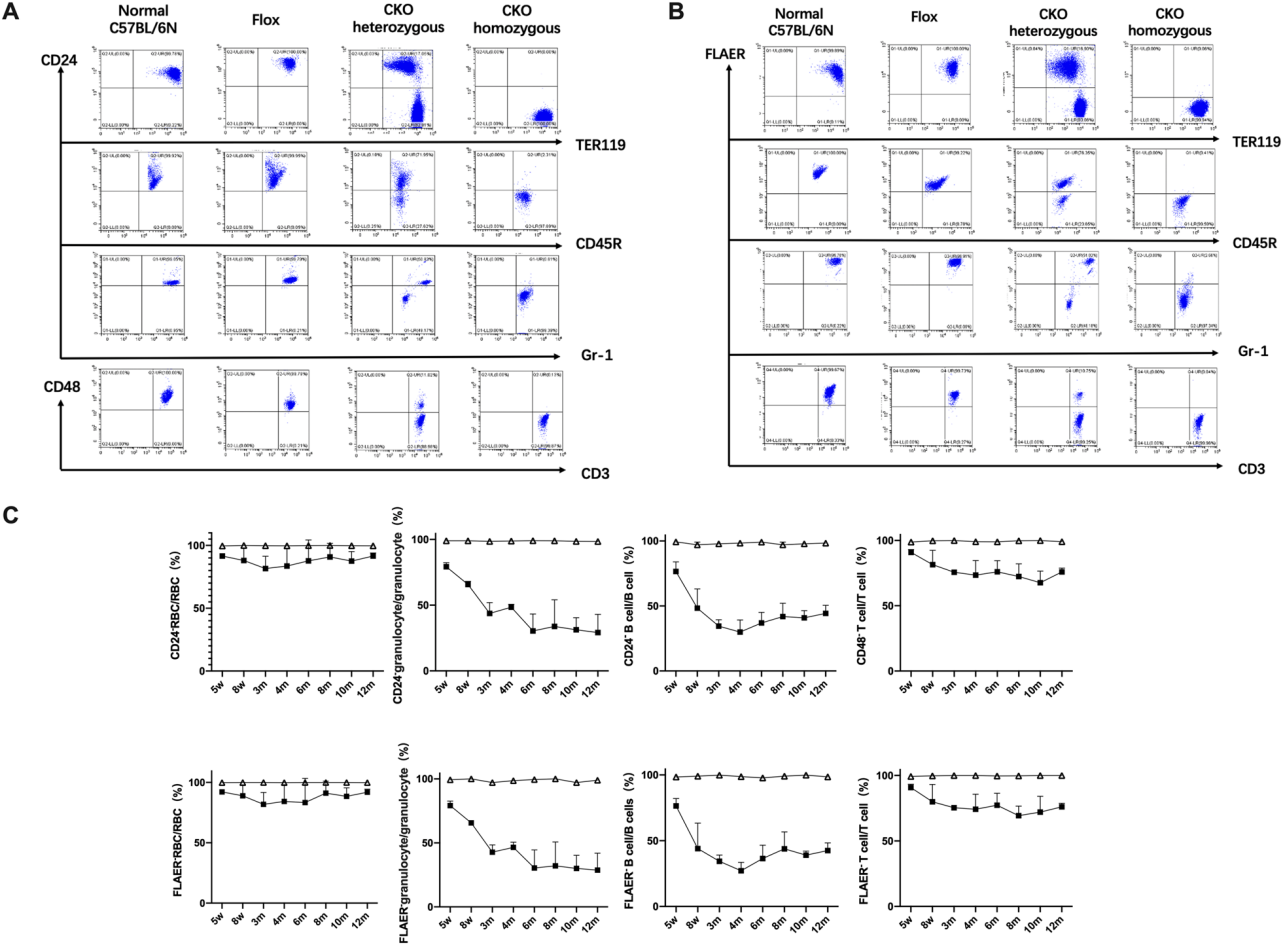
GPI and GPI-AP expression in peripheral blood cells of mice. **A** CD24/CD48 expression level. **B** FLAER expression level. **C** The change trend of GPI and GPI-AP deficiency proportion in peripheral blood cells of CKO mice (open triangle represents CKO homozygous, filled square represents CKO heterozygous))

The expression of GPI (FLAER) and GPI-AP (CD24/CD48) in different cell type (erythrocytes, granulocytes, B cells, and T cells) of peripheral blood cells were detected by FCM in four groups from 5w after birth and followed up to 1 year after birth. We detected the expression of FLAER on all cell types, detected the expression of CD24 on the surface of erythrocytes, granulocytes, B cells, and CD48 on T cells. The results showed that the expressions of GPI and GPI-AP in peripheral blood cells of CKO homozygous mice was almost completely absent. The proportion of the deficiency of GPI and GPI-AP in peripheral blood cells of CKO heterozygous mice was slightly different among different mice and different cell types, with the highest proportion of the deficiency in RBC and T cells, followed by B cells, and the proportion of the deficiency in granulocytes was about 20–30%. While the expressions of GPI and GPI-AP in peripheral blood cells of Flox mice and normal C57BL/6N mice were normal (Fig. 3A, B). With the prolongation of postnatal time, the deficiency proportion of GPI and GPI-AP in peripheral blood cells of homozygous CKO was stable. While the deficiency proportion in CKO heterozygous cells decreased gradually from birth until it reached a stable level at about 3 months after birth and remained there for life (Fig. 3C). Among them, the deficiency proportion of GPI on RBCs was always maintained at a high level.

**Fig. 4:**
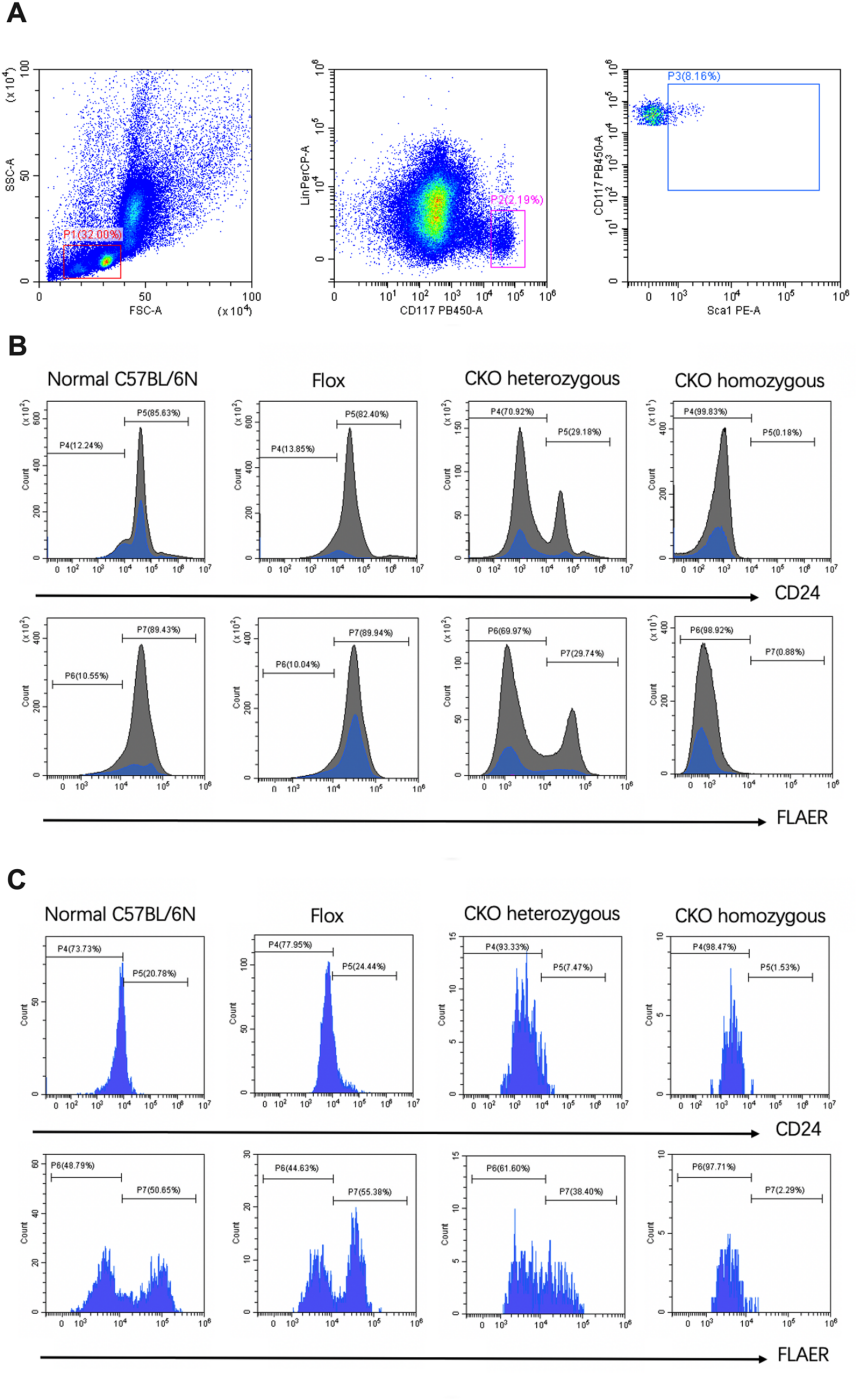
The expression levels of GPI and GPI-AP in bone marrow cells of mice. **A** P1 are the lymphocytes, P2 are the CD117^+^Lin^−^ cells in P1, P3 are Sca1^+^ cells in P2, and the P3 presented the hematopoietic stem cells which were labeled with Lin^−^CD117^+^Sca1^+^. **B** The expression levels of CD24 and FLAER in all bone marrow cells of mice (Grey) and P1 cells (Blue), P4 are the CD24^−^ cells, P5 are the CD24^+^cells, P6 are the FLAER^−^ cells, P7 are FLAER^+^ cells. **C** The expression levels of CD24 and FLAER in hematopoietic stem cells (P3)

We attempted to determine the reasons for the different GPI and GPI-AP deficiency proportion in different peripheral blood cell types of CKO heterozygous mice, so we detected the expression of FLAER and CD24 on bone marrow hematopoietic stem cells (HSCs). We used Lin^−^CD117^+^Sca1^+^ to label hematopoietic stem cells (Fig. 4A). The results shown that CD24/FLAER expression was grouped distinct and expressed uniformly in all bone marrow cells and lymphocytes (P1) (Fig. 4B). The expression of CD24 and FLAER on bone marrow HSCs (P3) in CKO homozygous mice was almost completely absent, while in Flox mice and normal C57BL/6N mice were only partially expressed. Besides, the expression levels of GPI and GPI-AP were inconsistent (Fig. 4C). We speculated that GPI and GPI-AP were not expressed in the whole course of hematopoietic cell differentiation, and the expression of early stem progenitor cells was incomplete. Although the proportion of the deficiency of CD24 and FLAER in bone marrow cells appeared to be greater in CKO homozygous than in CKO heterozygous, and greater in CKO mice than in Flox mice and normal mice, we did not compare the four groups later. Further research is needed to determine the reasons for the different GPI and GPI-AP deficiency proportion of different blood cell types.

In addition, we also found it interesting that FLAER can be used to detect the expression level of GPI in mouse erythrocytes, and the results were consistent with the expression level of GPI-AP. We detected FLAER and CD59 in peripheral blood erythrocyte of a healthy volunteer and a PNH patient, the results proved that FLAER cannot be detected in human erythrocytes (Additional file 1: Fig. S1).

### Pancytopenia was found in CKO homozygous mice, and leukopenia and anemia were found in CKO heterozygotes mice

**Fig. 5:**
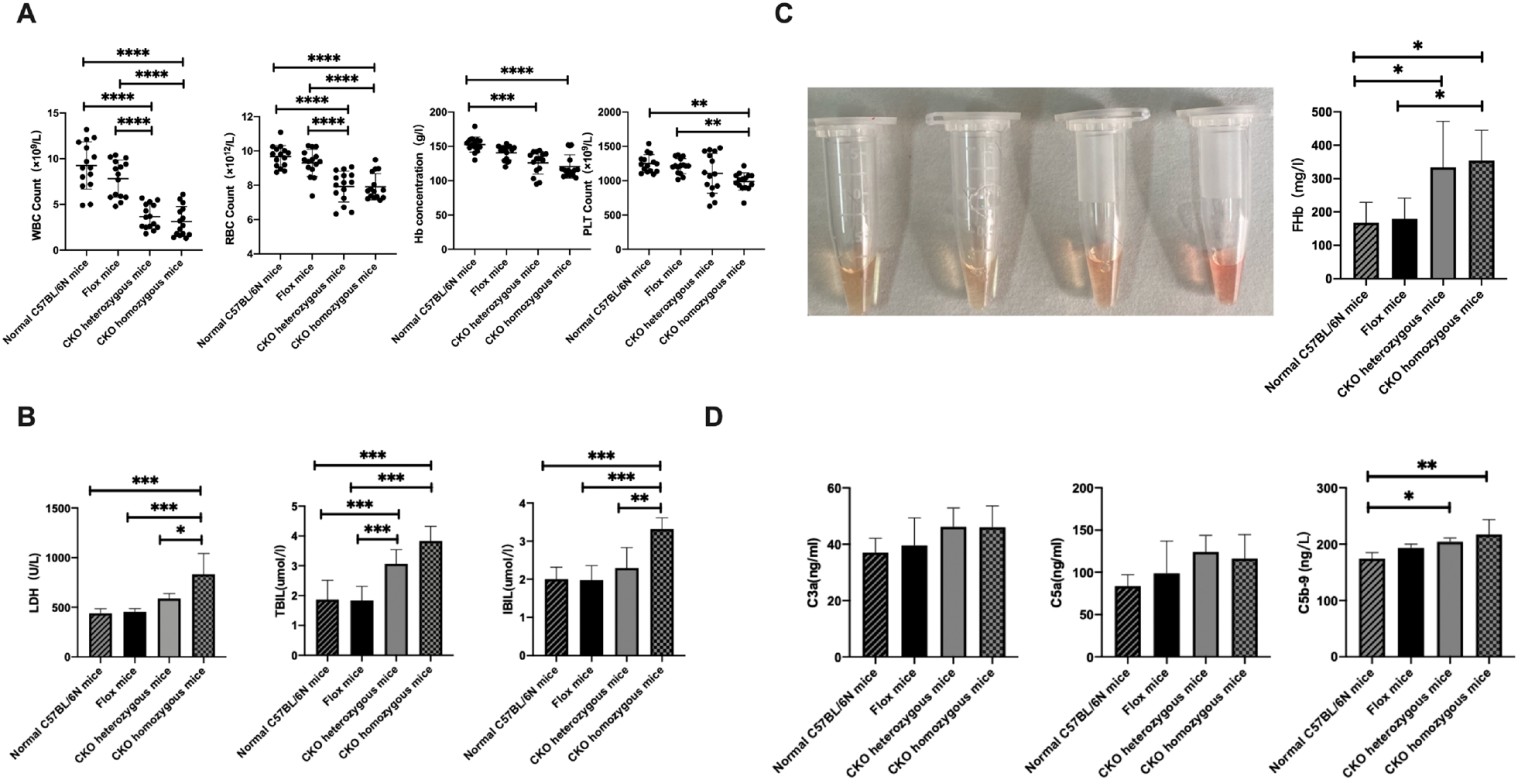
Blood cell count, hemolysis related indexes and complement level of mice. **A** Blood cell count. **B** LDH and Bilirubin levels. **C** Mice plasma color, from left to right, C57BL/6N mice, Flox mice, CKO heterozygous mice and CKO homozygous mice. **D** concentration of Serum C3a, C5a and C5b-9) (*p < 0.05, **p < 0.01, ***p < 0.001, ****p < 0.0001)

The WBC count, RBC count, Hb concentration and PLT count of the four groups were significantly different (*p* < 0.0001, *p* < 0.0001, *p* < 0.0001, *p* = 0.0008) (Table 3, Fig. 5A). We found pancytopenia in CKO homozygous mice, compared with normal C57BL/6N mice and Flox mice. While in CKO heterozygotes mice, only WBC and RBC counts were significantly lower than those in normal C57BL/6N mice (*p* < 0.0001, *p* < 0.0001) and Flox mice (*p* < 0.0001, *p* < 0.0001). The Hb concentration of CKO heterozygotes mice was lower than that of normal C57BL/6N mice (*p* = 0.0002). There were no statistical differences in the above indexes between CKO homozygous and CKO heterozygous mice, normal C57BL/6N and Flox mice.

**Table 3:** Laboratory data of mice

Indicators	Normal C57BL/6N mice	Flox mice	CKO heterozygous mice	CKO homozygous mice	*p*
WBC (× 10^9^/L)	9.260 ± 2.581	7.827 ± 2.025	3.667 ± 1.347	3.127 ± 1.637	< 0.0001*
RBC (× 10^12^/L)	9.679 ± 0.638	9.309 ± 0.790	7.923 ± 0.902	7.912 ± 0.765	< 0.0001*
Hb (g/L)	152.7 ± 11.07	140.6 ± 10.54	126.1 ± 16.64	120.6 ± 17.22	< 0.0001*
PLT(× 10^9^/L)	1249 ± 130.3	1213 ± 110.2	1106 ± 290.1	987.6 ± 126.9	0.0008*
LDH (U/L)	439.8 ± 45.11	453.1 ± 33.08	586.4 ± 51.83	832.9 ± 208.9	0.0001*
TBIL (μmmol/L)	1.868 ± 0.646	1.834 ± 0.475	3.064 ± 0.480	3.830 ± 0.493	< 0.0001*
IBIL(μmmol/L)	2.006 ± 0.308	1.974 ± 0.385	2.294 ± 0.539	3.318 ± 0.293	0.0032*
FHb (mg/L)	168.1 ± 61.00	179.8 ± 62.15	333.6 ± 137.7	354.1 ± 91.03	0.0052*
C3a (ng/mL)	37.05 ± 5.077	39.56 ± 9.813	46.21 ± 6.659	46.00 ± 7.639	0.1703
C5a (ng/mL)	83.80 ± 13.45	98.82 ± 38.01	124.2 ± 19.53	116.3 ± 28.45	0.1138
C5b-9 (ng/L)	174.3 ± 10.79	193.0 ± 7.10	204.1 ± 6.74	217.2 ± 26.39	0.0027*

^*^There was statistical difference between the four groups (*p* < 0.05)

### CKO homozygous mice has mild hemolysis

There were significant differences in serum LDH, TBIL and IBIL among the four groups (*p* = 0.0001, *p* < 0.0001, *p* = 0.0002) (Table 3, Fig. 5B). The serum LDH, TBIL and IBIL of CKO homozygous mice were significantly higher than those of normal C57BL/6N mice (*p* = 0.0002, *p* = 0.0001, *p* = 0.0004) and Flox mice (*p* = 0.0003, *p* = 0.0001, *p* = 0.0003). The serum TBIL level of CKO heterozygous mice was significantly higher than those of normal C57BL/6N mice (*p* = 0.0120) and Flox mice (*p* = 0.0098). The serum LDH and IBIL levels were higher in homozygous mice than that in heterozygous mice (*p* = 0.0140, *p* = 0.0041). There was no statistical difference in the above indexes between normal C57BL/6N mice and Flox mice.

We also observed plasma color of 4 groups, and found that CKO homozygous mice was the heaviest, CKO heterozygous was the second, and normal C57BL/6N mice and Flox mice were normal. The concentration of free hemoglobin (FHb) in plasma of the four groups was statistically different (*p* = 0.0052) (Fig. 5C). The level of FHb in CKO homozygous mice was higher than that in normal C57BL/6N mice and Flox mice (*p* = 0.0181, *p* = 0.0363), while the level in CKO heterozygous mice was only significantly higher than that in normal C57BL/6N mice (*p* = 0.0381). There was no statistical difference between CKO homozygous and CKO heterozygous mice, normal C57BL/6N mice and Flox mice.

The serum C5b-9 level of mice was significantly different among the four groups (*p* = 0.0027), in which CKO heterozygous and CKO homozygous mice were significantly higher than that of normal C57BL/6N mice (*p* = 0.0297, *p* = 0.0018). The serum C3a and C5a of CKO mice was no statistical difference between the four groups (*p* = 0.1703, *p* = 0.1138) (Fig. 5D).

Considering that complement activation in mice was relatively mild and the hemolysis phenotype was only chronic mild hemolysis, we attempted to use infection-activated complement to aggravate hemolysis or induce acute hemolysis in mice. But the CKO mice died soon (within 3 days) after nasal drip or gavage of bacterial liquid, while the normal C57BL/6N and Flox mice lived normally, which may be related to low white blood cells and poor anti-infection ability in CKO mice.

### Hemosiderin granulosa cells can be seen more easily in the spleens of CKO mice

**Fig. 6:**
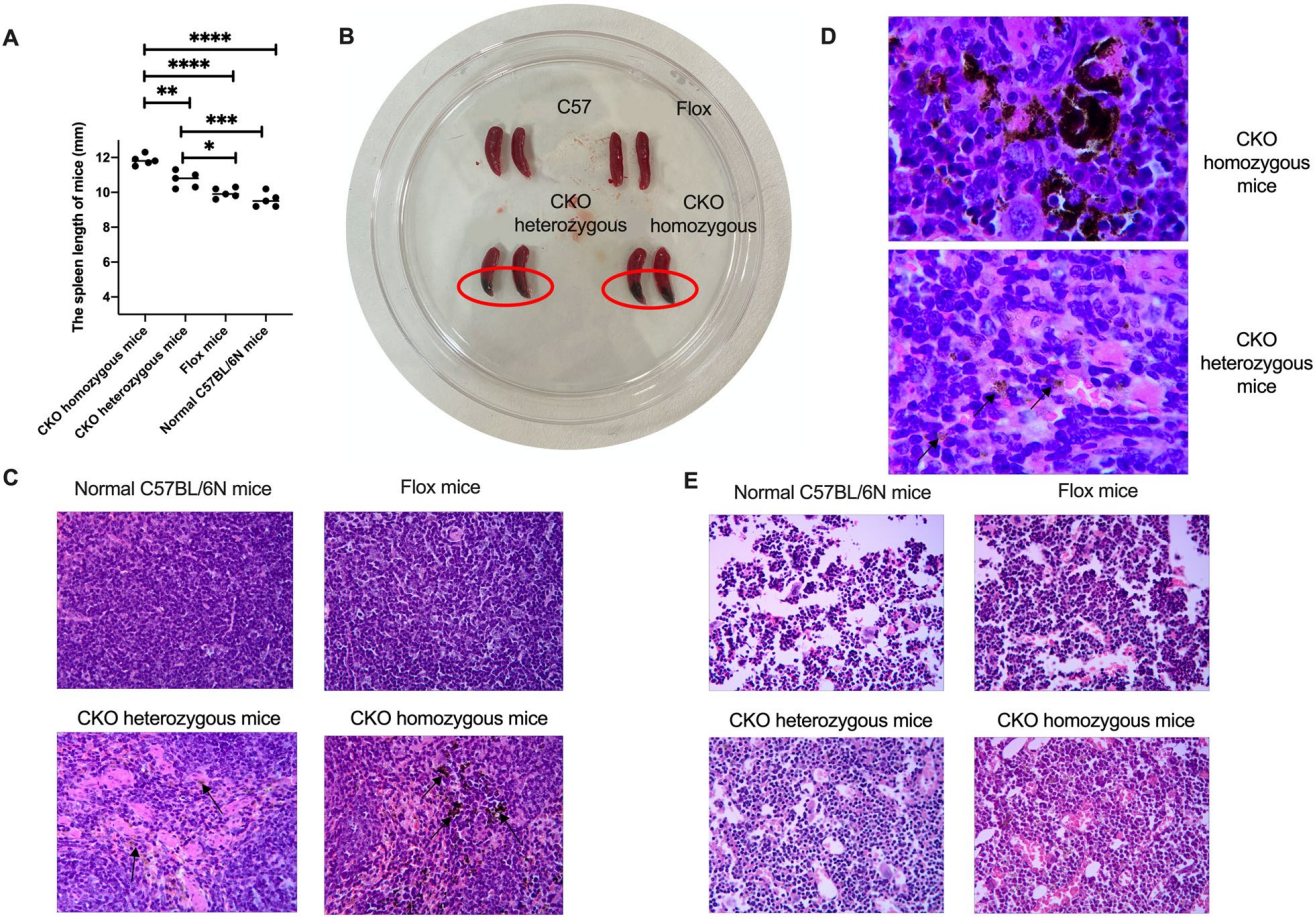
Pathology of the spleen and bone marrow. **A** The spleen length of four groups mice. **B** The spleen of four groups mice. **C** HE staining of spleen (10 × 40). **D** Hemosiderin in the cytoplasm of the hemosiderin granulosa cells (10×100). **E** HE staining of bone marrow (10 × 40)

20w CKO homozygous mice, CKO heterozygous mice, Flox mice and normal C57BL/6N mice were sacrificed. The spleen length (mm) of four groups was 11.84 ± 0.297, 10.72 ± 0.466, 9.96 ± 0.288 and 9.56 ± 0.416, (*p* < 0.0001) (Fig. 6A). What’s more, we can see nodules in the spleen of CKO mice (Fig. 6B). Spleen and bone marrow specimens of 4 groups were taken and HE staining was performed. The spleen structure of mice in the 4 groups was basically normal. Hemosiderin granulosa cells can be seen more easily in the spleens of CKO mice (Fig. 6C). We can see hemosiderin in the cytoplasm of the hemosiderin granulosa cells, and this type of cell in the spleens of CKO homozygous mice can be seen in small clusters and clumps (Fig. 6D). The granulocytes/erythrocytes% of bone marrow in the 4 groups were normal. Bone marrow hematopoietic tissue volume of normal C57BL/6N mice and Flox mice was about 90%VOL, and that of CKO heterozygous and CKO homozygous mice was about 95%VOL (Fig. 6E).

### Whole blood transcriptome sequencing analysis showed stable transcription levels in CKO mice

To more accurately understand the changes in CKO mice in addition to the *Pig-A* gene, RNA-seq was performed on CKO homozygous mice (Group A), CKO homozygous mice (Group B), Flox mice (Group C) and normal C57BL/6N mice (Group D). There were 3 6-month-old mice in each group, and they were named mice 1, 2 and 3, respectively. Before RNA-seq, flow cytometry was used to detect the expression levels of GPI and GPI-AP in blood cells of peripheral blood of 12 mice (Table 4). As mentioned above, the expression levels of GPI and GPI-AP in heterozygous mice were different in different mice and cell types. We found that the three heterozygous mice expressed similar GPI and GPI-AP expressions in red blood cells and T lymphocytes, but the proportion of GPI^−^ and GPI-AP^−^ cells in granulocyte and B lymphocytes of heterozygous B3 was significantly lower than that of B1 and B2 mice.

**Table 4:** Expression of GPI and GPI-AP in peripheral blood cells of 12 mice

	RBC		B cells		T cells		Granulocyte	
	CD24^+^	FLAER^+^	CD24^+^	FLAER^+^	CD48^+^	FLAER^+^	CD24^+^	FLAER^+^
A1	0	0	0.1	0	0.2	0	0.5	0.3
A2	0	0	0.5	0.3	0.4	0	1.4	0
A3	0	0	0	0.6	0	0.4	0.4	0
B1	2.39	2.36	54.11	55.92	22.15	22.15	37.70	33.02
B2	1.29	1.28	53.09	60.78	24.37	24.58	56.03	57.76
B3	5.18	5.10	73.30	75.41	26.82	26.22	86.0	85.6
C1	100	99.8	99.6	99.4	100	100	98.9	100
C2	99.8	100	100	100	98.9	99.2	100	99.4
C3	100	100	99.6	99.9	99.4	100	100	100
D1	99.6	99.4	100	99.2	99.3	100	100	99.6
D2	100	99.5	99.2	99.4	100	100	99.9	99.4
D3	99.9	100	99.2	100	99.2	99.2	100	99.3

A. CKO homozygous mice, B. CKO heterozygous mice, C. Flox mice, D. normal C57BL/6N mice

**Fig. 7:**
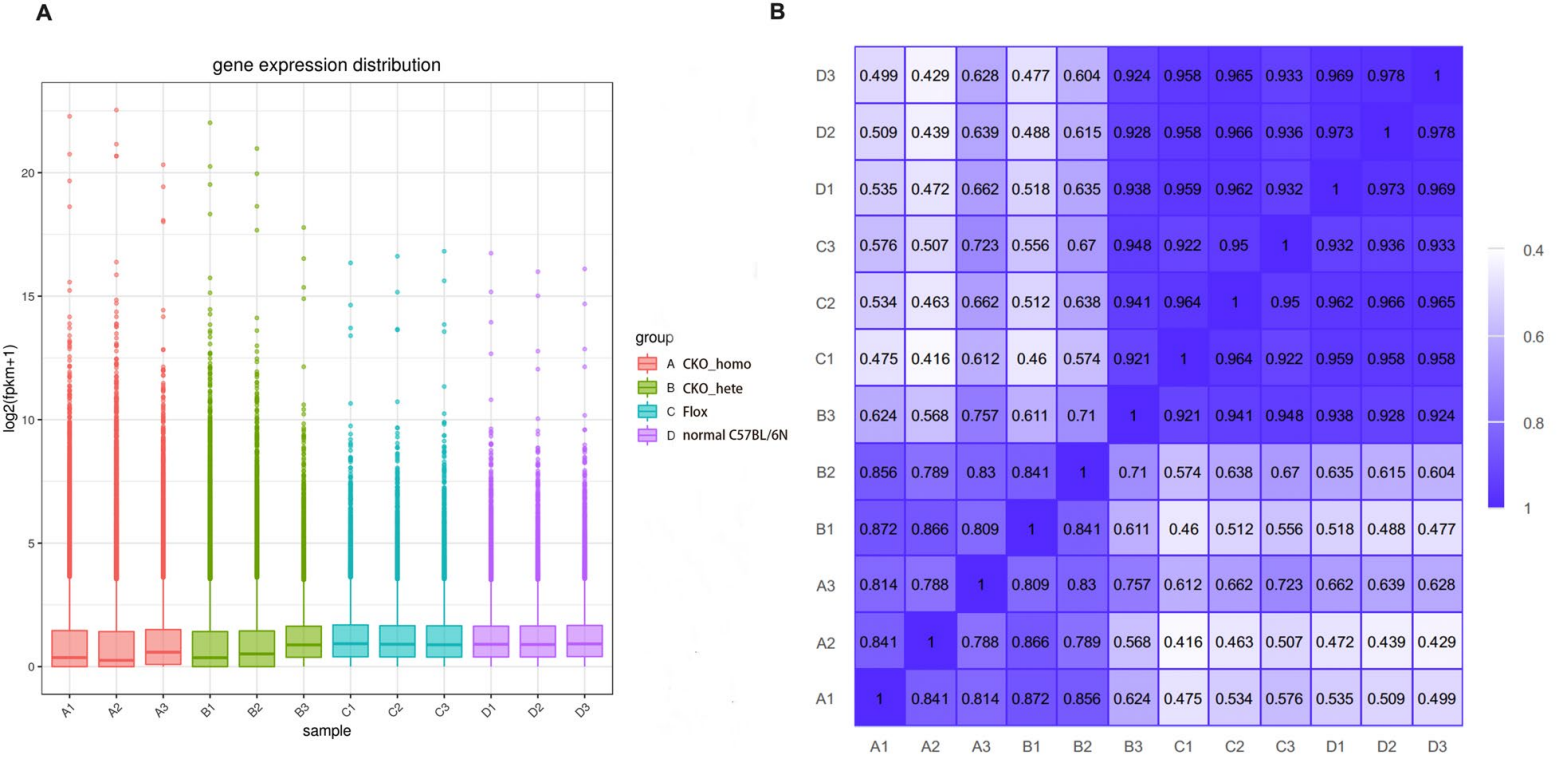
Distribution of gene expression levels in samples (**A**) and heat map of correlation between samples (**B**)

In RNA-seq, the 12 specimens generated 48,414,999 ± 3,568,965 raw_reads, and the error rate of each sample was less than 0.05. The percentage of G and C bases among the total number of bases (GC%) was 55.87 ± 1.318%. The percentage of bases with a Phred value greater than 20(Q20%) was 98.13 ± 0.2199%.After filtering, we end up with 45,961,537 ± 3,860,843 high-quality clean reads. We calculate the expression values of all genes in each sample by calibrated the sequencing depth and gene length using Fragments Per Kilobase Per Million Mapped reads (FPKM) (Fig. 7A). Furthermore, Pearson correlation coefficients of samples within and between groups were calculated according to FPKM values of all genes in each sample, so as to understand inter-group sample differences and intra-group sample duplication (Fig. 7B, Table 5). The closer the square of Pearson correlation coefficient (R^2^) is to 1, the closer the expression pattern is. Intra-group analysis results showed that the R^2^ of the four groups were all greater than 0.8. Interestingly, we found that the expression levels of B3 mice were more similar to Flox mice and normal C57BL/6N mice, which we speculated might be related to the low proportion of PNH clones in this mouse. Inter-group analysis results found that the R^2^ of Flox mice vs normal C57BL/6N mice was the highest (0.952 ± 0.014), followed by that of CKO homozygous mice vs CKO heterozygous mice (0.775 ± 0.108), and the R^2^ of heterozygous mice vs Flox/normal C57BL/6N mice was higher than that of homozygous mice vs Flox/normal C57BL/6N mice**.**

**Table 5:** Pearson correlation coefficient (R^2^) of intra-group analysis and Inter-group analysis

Intra-group analysis			
Group A	Group B	Group C	Group D
0.885 ± 0.101	0.817 ± 0.196	0.981 ± 0.016	0.962 ± 0.039

Inter-group analysis			
Group A vs Group B	Group A vs Group C	Group A vs Group D	
0.775 ± 0.108	0.552 ± 0.100	0.535 ± 0.088	

Group B vs Group C	Group B vs Group D	Group C vs Group D	
0.691 ± 0.194	0.681 ± 0.195	0.952 ± 0.014	

A. CKO homozygous mice, B. CKO heterozygous mice, C. Flox mice, D. normal C57BL/6N mice

**Fig. 8:**
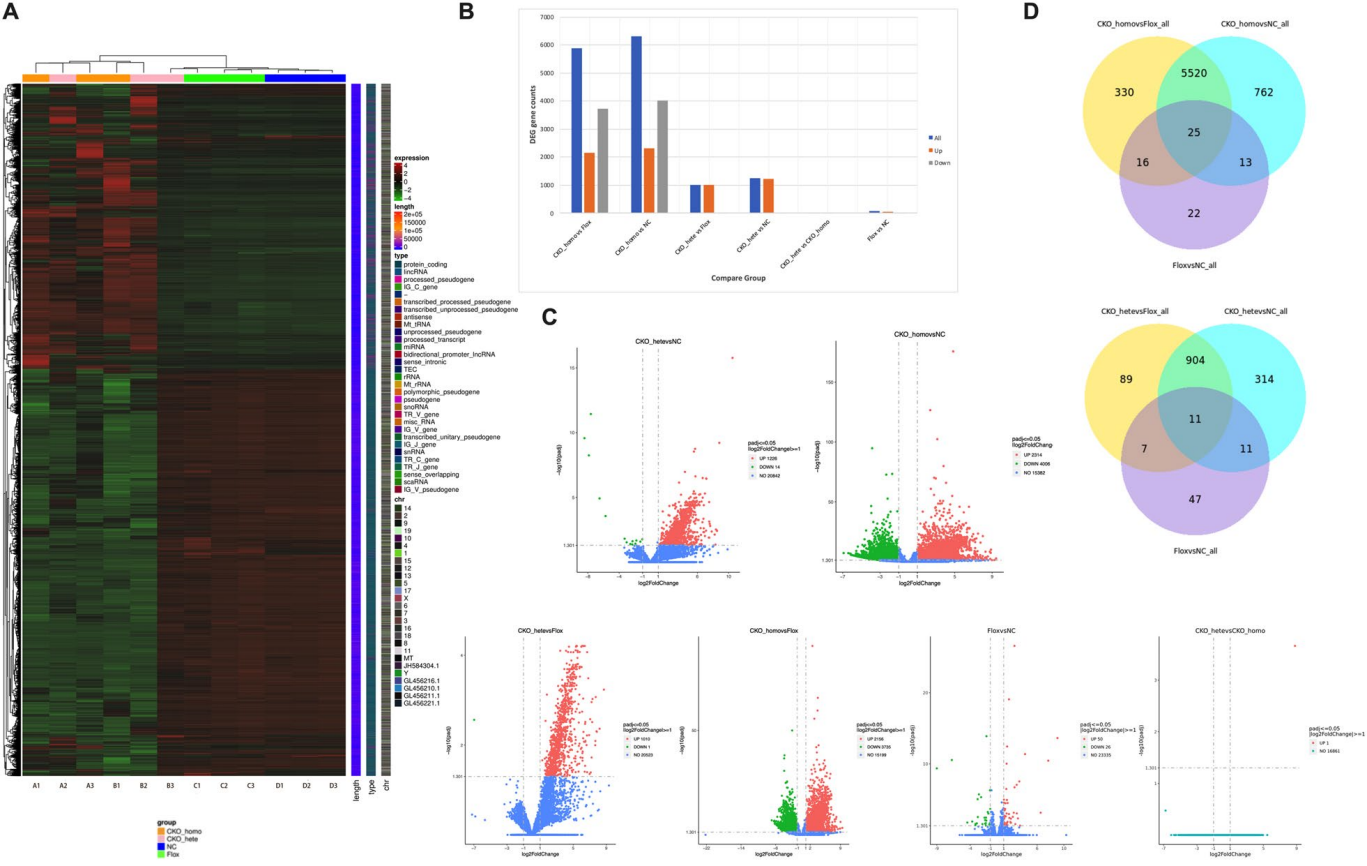
Differential analysis of gene expression. **A** Heat map of differentially expressed genes. **B** DEGs number statistics. **C** Volcanic map of DEGs comparison in each group. **D** Venn diagram of the overlap of different genes between different comparison combinations

After quantification of gene expression, we conducted statistical analysis on the expression data, and screened out differentially expressed genes (DEGs) with significant difference in expression level among different groups(|log2 (FoldChange) | ≥ 1 and *p* adj < 0.05). Heat map showed that DEGs expression was basically consistent in same group except B3, indicating stable transcription in CKO mice. Flox and normal C57BL/6N mice, CKO homozygous and CKO heterozygous mice expressions were basically similar (Fig. 8A). Consistent with the above-mentioned results of inter-group sample differences and intra-group sample duplication, the transcription levels of various genes in B3 mice are more similar to Flox and normal C57BL/6N mice, which may be related to its low PNH clones. The largest number of DEGs were compared between CKO homozygous and Flox/normal C57BL/6N mice, followed by CKO heterozygous and Flox/normal C57BL/6N mice (Fig. 8B, C). CKO homozygous mice were compared with Flox mice and normal C57BL/6N mice, respectively, and the two groups shared 5545 DEGs, including 25 DEGs that were also detected in Flox mice and normal C57BL/6N mice. When compared CKO heterozygotes mice with Flox and normal C57BL/6N mice, respectively, we found 915 shared DEGs, including 11 DEGs that were also detected in Flox and normal C57BL/6N mice (Fig. 8D).

**Fig. 9:**
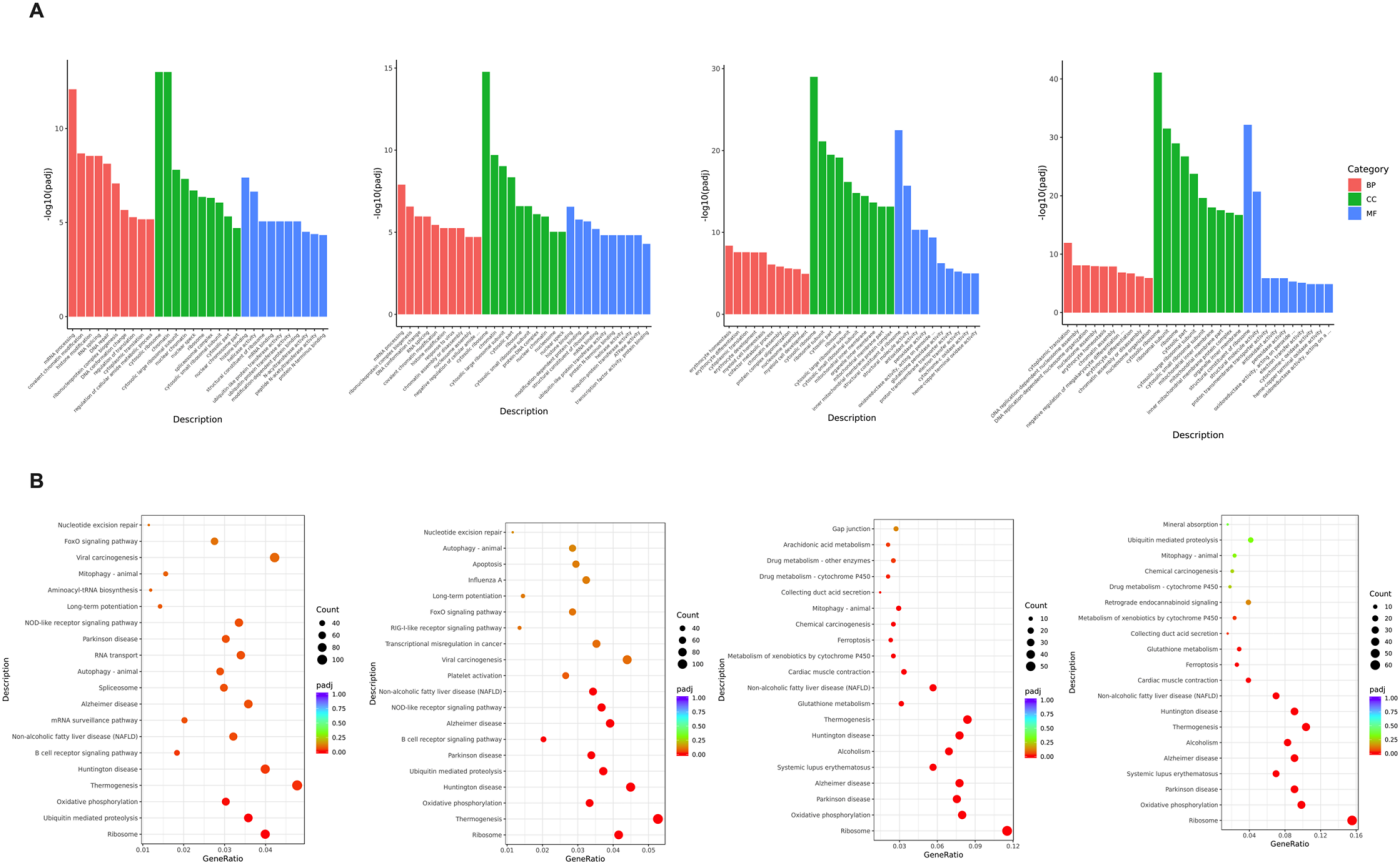
Functional enrichment analysis of DEGs. **A** GO enrichment analysis. **B** KEGG pathway enrichment analysis. From left to right, CKO homozygous vs Flox mice, CKO homozygous vs normal C57BL/6N mice, CKO heterozygous vs Flox mice, CKO heterozygous vs Normal C57BL/6N mice

Considering the similar expression patterns of CKO homozygous and CKO heterozygous mice, Flox and normal C57BL/6N mice, we only selected the DEGs of CKO mice and Flox/normal C57BL/6N mice for gene function enrichment analysis (Fig. 9). Regardless of Go enrichment analysis or KEGG pathway enrichment, the comparison between CKO mice and Flox mice was basically consistent with that between CKO mice and normal C57BL/6N mice, which also proved that the simple insertion of LoxP site did not affect the transcription pattern of mice. Comparison between CKO homozygous mice and Flox/normal C57BL/6N mice showed that DEGs enriched Go-BP was mostly related to mRNA process, ribosome protein synthesis and epigenetic modification. Go-CC was enriched in ribosome and its components, while Go-MF was enriched in ribosome structural composition and combination of histone and mRNA. Comparison between CKO heterozygous and Flox/normal C57BL/6N mice showed that DEGs enriched Go-BP was mostly related to RBC differentiation, development, cellular homeostasis and epigenetic modification. Go-cc was enriched in ribosomes and their components, while GO-MF was enriched in ribosomes assembly and activation of structural molecules (Fig. 9A). The most significant enrichment of KEGG pathway in all four groups was in ribosome related pathway (Fig. 9B).

## Discussion

PNH is a benign clonal disease caused by hematopoietic stem cell *PIG-A* gene mutation, which is a common cause of intravascular hemolysis. *PIG-A* gene encodes an important enzyme in the process of GPI synthesis [16]. Mutation of *PIG-A* gene leads to abnormal GPI synthesis, and GPI deficiency leads to the inability of GPI-AP (such as CD16, CD55, CD9, etc.) to connect to cell membranes, causing complement activation and blood cell destruction [17, 18]. *PIG-A* mutations in somatic cells of PNH patients are varied, and more than hundreds of types have been reported. No hot spot mutations have been found. Although the *PIG-A* gene mutation can also be detected in normal people, it is a polyclone originating from the stage of directed hematopoietic progenitor cells [19], which does not have the ability of self-renewal and can only survive for 3–4 months, so it will not develop disease. While *PIG-A* gene mutations in PNH patients originated from the hematopoietic stem cell stage and were monoclonal, or there were multiple mutations, but there was a dominant mutation gene [16].

Although *PIG-A* mutation is the initiator of PNH, certain specific factors lead to abnormal PNH clones in hematopoietic stem cells with *PIG-A* mutation, and abnormal PNH clones and normal hematopoiesis coexist in patients. Later, due to the action of immune factors and other factors, abnormal PNH clones obtained proliferation advantages, leading to the pathogenesis of PNH [20, 21]. At present, there are three theories about how abnormal PNH clones obtain proliferation advantage, including immune escape [22, 23], anti-apoptotic mechanism [24] and secondary gene mutation [25–27]. However, due to the lack of mature disease model, further research is still lacking. The animal models of PNH reported in previous literature, whether mouse model or rhesus monkey model, have abnormal PNH clones, but they all lack disease phenotypes such as anemia, hematuria, thrombosis, etc. [8–14], so they cannot be really used in the study of PNH. It is very urgent for us to establish a hemolytic PNH model. Therefore, we tried to construct a mouse model of *PIG-A* gene knockout in hematopoietic system specifically mediated by Vav-iCre [28], which has not been used in previous PNH models. We believe that specific *Pig-a* gene knockout in hematopoietic cells may be more able to match the characteristics of clinical PNH patients. In our study, Flox mice were constructed by inserting a codirectional loxP locus on each side of exon 3 and exon 5 of *Pig-a* gene in embryonic stem cells of C57BL/6N mice. Flox mice were then mated with Vav-iCre to produce a *Pig-a* gene knockout mouse in hematopoietic system (CKO mice).

After identification, we found that all blood cells of CKO homozygous mice were completely deficient in GPI and GPI-AP, which can always be completely knocked out in the presence of only abnormal clone cells. While there were both normal hematopoiesis and abnormal clones in the CKO heterozygous mice. Interestingly, the proportion of GPI and GPI-AP deficiency gradually decreased as the CKO heterozygous mice grow older, especially in granulocytes and lymphocytes, and stabilized at about 3 months and maintained for life. This result was not the same as that of PNH patients. Sequencing of 23 PNH patients in our center found that most female PNH patients had *PIG-A* heterozygous mutation, but the proportion of PNH clones was stable or gradually increased. The differences between the patient and PNH mice models also reconfirmed that the proliferation advantage of PNH clones requires the participation of other unknown factors besides the *PIG-A* mutation. Some scholars have studied the secondary mutation genes of PNH patients and screened out some high-frequency mutations [29–31], while the mechanism research is still very difficult. The CKO heterozygous mice might be the suitable model for the further study. Another interesting thing in CKO heterozygous mice is that the percentages of GPI deletion were not similar in different cell lines, with the highest in erythroid cells (about 80%) and the lowest in granulocyte and B lymphocytes (about 30%). As we all know, in PNH patients, GPI deletion is highest in granulocytes, lowest in lymphocytes, which we cannot explain the mechanism. Deeper study on immunological function in CKO heterozygous mice maybe continued in the further to help us explain more mechanism in PNH.

In addition to having stable GPI and GPI-AP deficient, we also demonstrated intravascular hemolysis in our mouse models. We detected more hemolysis-related indicators not involved in previous mouse models, including serum LDH, TBIL, IBIL, complement C5b-9 levels, and FHb. Fortunately, mild hemolysis and mild hypocytosis were found in CKO mice, especially homozygous CKO mice. CKO mice showed mild hemolysis, and the deposition of hemosiderin granulosa cells in the spleen suggested that intravascular hemolysis in this model mouse was a long-term and chronic process. Furthermore, CKO mice had an increase in the volume of bone marrow hematopoietic tissue, which we believed to be a compensative reaction. We believe that the mild and chronic hemolysis and the existence of compensatory mechanism in vivo are the reasons for the normal survival of CKO mice. Unfortunately, all disease markers in CKO mice showed only mild and chronic intravascular hemolysis. We tried to use infection-activated complement to aggravate hemolysis or induce acute hemolysis in mice, but the mice died soon after intervention, which may be related to low white blood cells and poor anti-infection ability in mice. We will further optimize or try something else at a later stage.

Finally, RNA-seq was performed on CKO mice, Flox mice and normal C57BL/6N mice to determine the effect of Pig-A gene knockdown on the transcription level of mice, and to provide background information for subsequent functional tests of other disease-related influencing factors using this mouse model. The results showed that Flox mice with loxP alone were similar to normal C57BL/6N mice, which proved that the simple insertion of LoxP site did not affect the transcription of mice. In addition, the results showed that CKO mice have stable transcription characteristics and are ideal model animals. DEGs of CKO homozygous mice vs Flox mice, CKO homozygous mice vs normal C57BL/6N mice, CKO heterozygous mice vs Flox mice, CKO heterozygous mice vs normal C57BL/6N mice were compared, and the results showed that the DEGs in the four groups were all enriched in ribosomes and their components and enriched in ribosomal related pathways. These results indicate that the knockout of PIG-A gene mainly affects protein synthesis, which may be related to the fact that the protein product encoded by PIG-A gene is an important enzyme required for the first step of GPI biosynthesis. In addition, we found that some differential genes in CKO homozygous mice were enriched in RNA splicing, DNA repair, histone modification and DNA conformation change. We speculated that this result might indicate that PIG-A gene knockout may be involved in the pathogenesis of PNH through epigenetic regulation, and we will verify this hypothesis through further experiments in the future. We found that one CKO heterozygous mouse(B3) had similar transcriptional characteristics to Flox/normal C57BL/6N mice, and this mouse had a lower PNH clones of B cells and granulocyte than the other two heterozygous mice. In addition, the results of differential gene enrichment showed that some of the differential genes in CKO heterozygous mice and Flox/normal C57BL/6N mice were enriched in erythrocyte homeostasis, erythrocyte differentiation and development, which may be the reason why the proportion of red PNH clones in CKO heterozygous mice can always be maintained at a high level, but the specific mechanism is still unclear. Therefore, when using CKO heterozygous mice for subsequent experiments related to the clone’s ratio, we suggest selecting CKO heterozygous mice older than 3 months with similar and greater than 50% PNH clone proportion of B cells and granulocyte.

In general, we successfully constructed a *Pig-a* conditional knock-out mice model mediated by Vav-iCre with not only GPI deficient but also mild hemolysis. CKO homozygous mice have invascular hemolysis, and only abnormal PNH abnormal cloning which were stable for life. Although the hemolytic phenotype of CKO heterozygous mice was lighter than that of CKO homozygous mice, both abnormal clones and normal hematopoiesis existed in CKO heterozygous mice, and the proportion of abnormal PNH clones could reach a stable level at about 3 months of birth and maintain for life. CKO mice have stable transcription characteristics, and the characteristics of CKO homozygous mice and CKO heterozygous mice are different, which is suitable for different studies in the future. CKO homozygous mice are suitable for studying the therapeutic effect of new drugs, while CKO heterozygous mice are more suitable for studying the abnormal clonal proliferation advantage of PNH.

## Conclusion

We constructed a hematopoietic system-specific *Pig-a* gene knockout mice (CKO mice) using ES targeting and Vav-iCre. It was verified that the mRNA and protein expression levels of Pig-a in the hematopoietic system of CKO mice were knocked down, and the expression of GPI and GPI-AP were absent in peripheral blood cells of CKO mice. In CKO mice, anemia, mild increase of LDH, TBIL and IBIL, increased level of free hemoglobin and complement C5b-9, and increased number of hemosiderin granules in spleen suggested the presence of intravascular hemolysis, indicating the successful establishment of a *Pig-a* conditional knock-out mice model mediated by Vav-iCre which had stable GPI-deficient and mild hemolysis.

## Supplementary Information

**Additional file 1: Figure S1.** CD59 and FLAER expression levels in erythrocytes (A a healthy volunteer, B a PNH patient).

## Data Availability

The datasets during and/or analyzed during the current study available from the corresponding author on reasonable request.
