# Supplementary figures and images for: A Pig-a conditional knock-out mice model mediated by Vav-iCre: stable GPI-deficient and mild hemolysis
Source: Exp Hematol Oncol. 2022 Jan 15;11:1. doi: 10.1186/s40164-022-00254-5 (PMC8760646; doi:10.1186/s40164-022-00254-5)

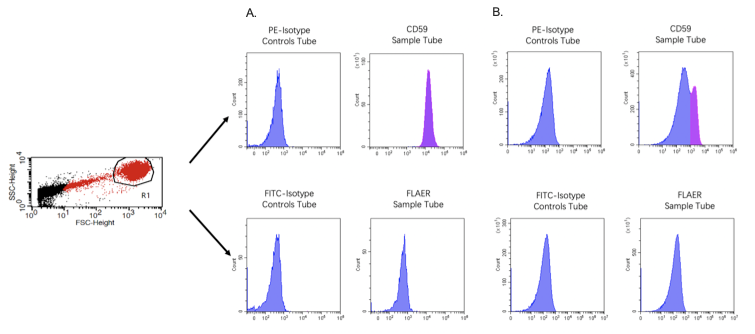


Figure S1. CD59 and FLAER expression levels in erythrocytes

(A. a healthy volunteer, B. a PNH patient)

Supplement: Supplementary file 1 — Additional file 1: Figure S1. CD59 and FLAER expression levels in erythrocytes (A a healthy volunteer, B a PNH patient). [file 40164_2022_254_MOESM1_ESM.docx]
